# Observation of distorted tilted conical phase at the surface of a bulk chiral magnet with resonant elastic x-ray scattering

**DOI:** 10.1080/14686996.2025.2532366

**Published:** 2025-07-15

**Authors:** S. Mehboodi, V. Ukleev, C. Luo, R. Abrudan, F. Radu, C. H. Back, A. Aqeel

**Affiliations:** a School of Natural Sciences, Technical University of Munich, Garching, Germany; b Munich Center for Quantum Science and Technology (MCQST), Munich, Germany; c Center for Quantum Engineering (ZQE), Technical University Munich, Garching, Germany; d Helmholtz-Zentrum Berlin für Materialien und Energie, Berlin, Germany; e Institute of Physics, University of Augsburg, Augsburg, Germany

**Keywords:** Resonant elastic X-ray scattering, magnetic structure, distorted tilted conical, multidomain low-temperature skyrmion

## Abstract

We report on various magnetic configurations including spirals and skyrmions at the surface of the magnetic insulator Cu${\;}_{2}$OSeO${\;}_{3}$ at low temperatures with a magnetic field applied along 100 using resonant elastic X-ray scattering (REXS). We observe a well-ordered surface state referred to as a distorted tilted conical spiral (dTC) phase over a wide range of magnetic fields. The dTC phase shows characteristic higher harmonic magnetic satellites in the REXS reciprocal space maps. Skyrmions emerge following static magnetic field cycling and appear to coexist with the dTC phase. Our results indicate that this phase represents a distinct and stable surface state that does not disappear with field cycling and persists until the field strength is increased sufficiently to create the field-polarized state.

## IMPACT STATEMENT

We observed a well-ordered surface texture referred to here as distorted titled conical spiral phase in an extended field region of the magnetic phase diagram of the B20 skyrmion hosting material Cu_2_OSeO_3_ at low temperatures that shows characteristic higher harmonic magnetic satellites in the REXS reciprocal space maps and coexists with the multidomain skyrmion phase populated by the field cycling protocol.

## GRAPHICAL ABSTRACT

**Figure:**
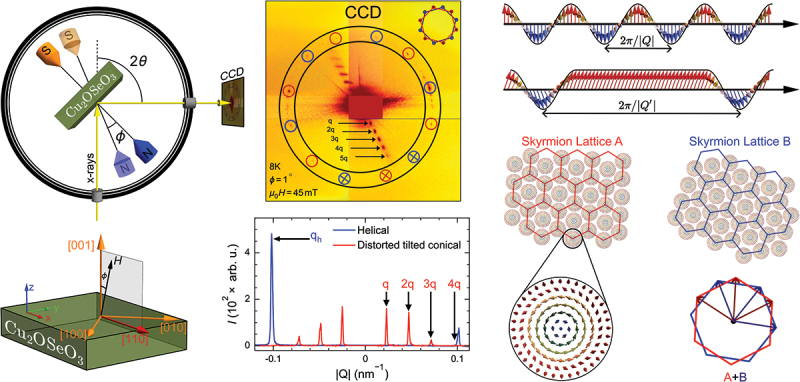

## 1. Introduction

In recent years, cubic chiral magnets have gained significant interest for their ability to host unique chiral magnetic spin configurations like helical spirals, chiral soliton lattices [1,2], skyrmions [3–5], screw dislocations and tilted spiral [6,7]. These noncollinear spin configurations are largely driven by the antisymmetric Dzyaloshinskii-Moriya interaction intrinsic in these chiral magnets, offering new avenues for emerging computing technologies [8–10]. The topologically nontrivial skyrmion textures were initially observed in the cubic chiral magnet MnSi using neutron scattering techniques [3] and subsequently in Cu${\;}_{2}$OSeO${\;}_{3}$ [5]. The latter is a unique member of the cubic chiral magnet family because it is an insulator with a wide band gap of 2.4 eV [11] providing the opportunity to control the skyrmion lattice phase by electric fields [12,13]. In addition, the low Gilbert damping [14,15] observed in Cu${\;}_{2}$OSeO${\;}_{3}$ makes it promising for potential applications in magnonic devices [16]. Cu${\;}_{2}$OSeO${\;}_{3}$ hosts two independent skyrmion pockets at different magnetic field-temperature regions of the phase diagram [17–19]. It has been proposed, that the competition between cubic magneto-crystalline and exchange anisotropies in Cu${\;}_{2}$OSeO${\;}_{3}$ at low temperatures [20] leads to the stabilization of novel noncollinear magnetic phases, such as the tilted conical state and the low-temperature skyrmion lattice phase. Notably, these phases are only observed when the magnetic field is applied along the crystallographic $\left\langle 100\right\rangle$ directions [17,18,21,22].

In chiral magnets, the spin configurations at the surface can significantly differ from those in the bulk due to the lack of translational symmetry, anisotropy, and surface Dzyaloshinskii–Moriya interaction [23]. These factors may cause surface twists that change the helicity angle of the spin configurations at the surface compared to the bulk [24–29]. Recent investigations highlight the surface twists of spirals and skyrmion phases in Cu${\;}_{2}$OSeO${\;}_{3}$ [24,25]. Here, we focus on the spiral and skyrmion phases at the surface of the cubic chiral magnet Cu${\;}_{2}$OSeO${\;}_{3}$ that exist at low temperatures by applying a magnetic field along the $\left\langle 100\right\rangle$ crystallographic directions. We investigate these magnetic phases by using resonant elastic X-ray scattering. REXS enables the identification of various magnetic phases through reciprocal phase mapping using element-specific X-ray energies [30]. In the case of the B20-type cubic chiral helimagnets, the crystal’s small lattice constant relative to the X-ray wavelength leads to the exclusion of structural peaks in diffraction according to Bragg’s law. Conversely, in the Cu${\;}_{2}$OSeO${\;}_{3}$ crystal with a comparatively large lattice constant [31] among the B20 members, a forbidden peak emerges in soft X-ray measurements at the Cu L-edge. By employing REXS and characterizing the modulated magnetic orders with the presence of magnetic satellites surrounding the Bragg peaks, one can effectively map magnetic phases in reciprocal space. This comprehensive approach enables a detailed investigation of the magnetic phases of Cu${\;}_{2}$OSeO${\;}_{3}$.

**Figure 1.**
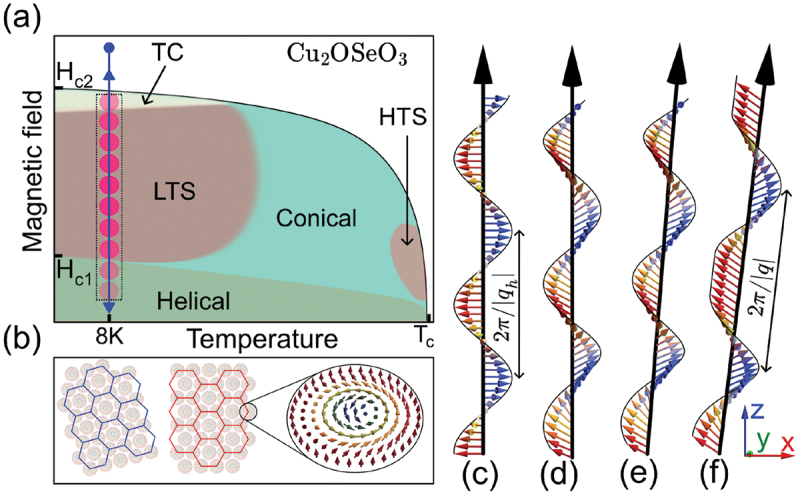
(a) Schematic illustration of the magnetic phase diagram for Cu${\;}_{2}$OSeO${\;}_{3}$ for magnetic field applied along $\left\langle 001\right\rangle$. HTS, LTS, and TC indicate high-temperature skyrmion, low-temperature skyrmion, and tilted conical spiral phases. The REXS measurements were performed exclusively in the field region indicated by the blue arrow at T≈ 8 K, and the phase boundaries are shown for illustration purposes only and were not determined from our measurements. (b) Shows a schematic representation of two differently oriented hexagonal skyrmion lattices (blue and red) featuring Bloch-type skyrmions. (c), (d), (e), and (f) shows the magnetic configurations of helical, conical, TC, and dTC spirals, respectively.

Figure 1 presents a schematic illustration of the main magnetic phases of a bulk Cu${\;}_{2}$OSeO${\;}_{3}$ single crystal when a magnetic field is applied along the $\left\langle 100\right\rangle$ crystallographic direction. Below the critical temperature $T_{c}$, Cu${\;}_{2}$OSeO${\;}_{3}$ favors helimagnetic long-range order in a large region of the phase diagram [5,32], characterized by a helical pitch, $\lambda_{h}$, and wave vector $q_{h}$ (Figure 1(c)) [33]. The balance between magneto-crystalline cubic anisotropy and the anisotropic exchange terms determines the helical spiral orientation along the easy anisotropy direction [5] which are $\left\langle 100\right\rangle$ and $\left\langle 111\right\rangle$ for Cu${\;}_{2}$OSeO${\;}_{3}$ and MnSi, respectively. When a finite magnetic field H is applied, it aligns the helix axis and tilts the magnetic moments towards the field direction giving rise to a conical magnetic phase retaining the same pitch $\lambda_{h}$ (see Figure 1(c,d)).

At large applied magnetic fields $H\geq H_{c2}$, all magnetic moments fully align along the applied magnetic field direction, resulting in the establishment of a field-polarized state. Close to $T_{c}$, a skyrmion lattice is observed in a narrow field-temperature region, referred to here as the HTS phase. The HTS is not only observed in Cu${\;}_{2}$OSeO${\;}_{3}$ but is also inherent in other cubic helimagnets like Fe${\;}_{1-x}$Co${\;}_{x}$Si [34], MnSi [3] and FeGe [35]. It is stabilized by thermal fluctuations and as a result observed at elevated temperatures near $T_{c}$. Recently, a tilted conical spiral (TC) [21] and another skyrmion lattice phase (referred to here as low-temperature skyrmion phase – LTS) [17] were observed to be stabilized due to cubic crystalline and exchange anisotropy contributions at low temperatures when the external magnetic field is applied along the $\left\langle 100\right\rangle$ crystallographic direction. Note that the LTS is an independent skyrmion lattice phase and compared to thermodynamically stabilized HTS [17,22] it is nearly isotropic for magnetic fields applied along different crystallographic directions. Furthermore, at relatively high magnetic fields, the spiral orientation deviates from alignment with the magnetic field direction, characterized as a TC phase originating from the competition between cubic anisotropy with $\left\langle 100\right\rangle$ easy axis and exchange anisotropy with $\left\langle 111\right\rangle$ easy axis, specifically when the field is close to $H_{c1}$ (Figure 1(e)) [21]. The transition from a field-polarized state to a topologically non-trivial LTS phase is rather complicated and the system goes through a TC phase before settling in a LTS phase. The LTS and TC phases are strongly hysteretic and depend on the field sweep directions.

In this work, we experimentally detect an orientationally ordered skyrmion phase by applying a magnetic field along the $\left\langle 100\right\rangle$ direction by following the cycling field protocol at low temperatures. Most interestingly, surface effects lead to the unexpected formation of a robust dTC spiral phase (Figure 1(f)) that persists throughout the field cycling process used to populate skyrmions in the LTS phase.

## 2. Experimental

REXS experiments are performed using the ALICE 2 endstation, at the PM3 beamline at BESSY 2 synchrotron, Berlin. Cu${\;}_{2}$OSeO${\;}_{3}$ single crystals were grown by chemical vapor transport [36] and then oriented with a Laue diffractometer. One Cu${\;}_{2}$OSeO${\;}_{3}$ crystal was precisely cut into a cuboid with 3×2 mm${\;}^{2}$ lateral dimensions and 1 mm thickness. The crystal was oriented with a surface normal along $\left\langle 100\right\rangle$ and edges along $\left\langle 110\right\rangle$ crystallographic directions and then the top surface was polished mechanically [37]. The sample was mounted on a copper sample holder, with the polished surface facing upward. The sample holder includes a cylindrical temperature shield with a 210${\;}^{\circ })$ cut open arc of 2 mm width that enables a free path for incoming and outgoing X-ray beams. All REXS results presented in this paper were acquired at the L${\;}_{3}$ edge using a photon energy of 931.8 eV (λ≈ 1.33 nm) and circularly polarized X-rays, which probe the magnetization component parallel to the incident beam. The forbidden structural Bragg peak at approximately $2\theta \;\;=96.5^{\circ }$ is observed exclusively in resonant X-ray scattering [33,38]. Resonant X-ray scattering is sensitive to the local point group symmetry, and thus the symmetry of the measurement can sometimes break the general symmetry of the crystal, including features like glide planes or screw axes that usually cause certain reflections to extinct. In the case of Cu${\;}_{2}$OSeO${\;}_{3}$, the (001) reflections are typically forbidden by the screw-axis symmetry. Their appearance in REXS measurements can be explained by the anisotropy of the tensor of X-ray susceptibility, which can allow these reflections and their magnetic satellites to become visible. Fortunately, due to the large lattice constant of the crystal (ca. 0.8925 nm [31]) the (001) reflection is accessible at the Cu L-edge.

**Figure 2.**
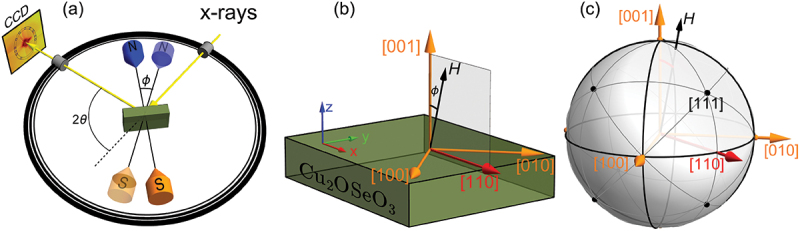
(a) Schematic representation of the experimental setup. The X-ray beam is scattered off the sample satisfying the 2θ=96.5${\;}^{\circ }$ diffraction condition and is captured by a CCD camera. ϕ represents the rotational angle of magnetic field w.r.t to the sample normal ($\left\langle 001\right\rangle$) (b) and (c) highlight the coordinate system of field orientation relative to crystallographic directions of the sample.

The REXS intensity was collected by the 4k CCD detector (GreatEyes GmbH, Germany) located at a distance of 73 cm from the sample [39]. The intense (001) Bragg peak was blocked by a beamstop, allowing to observe the magnetic satellites. In this manuscript, all results are obtained at a base temperature of approximately 8 K, utilizing the closed-cycle cryostat (Stinger, ColdEdge Technologies, USA). To improve the signal-to-noise ratio, we averaged ten images, each measured at 20 seconds exposure time, and then subtracted the background at $\mu_{0}H$ = 150 mT (field polarized state). We conducted our measurements by tilting the magnetic field from the $\left\langle 001\right\rangle$ crystallographic direction towards the $\left\langle 110\right\rangle$ direction, denoted as angle ϕ (Figure 2), with a maximum tilt angle of 11 degrees. This adjustment was performed to enable a detailed study of the dTC phases and their behavior under varying magnetic field strengths.

## 3. Results

REXS revealed the existence of a periodic anharmonic magnetic order, identified as a dTC spiral phase (see Figure 1(f)), in addition to the proper screw characteristic of the helical spirals in the chiral helimagnetic material Cu${\;}_{2}$OSeO${\;}_{3}$. As shown in Figure 1(f), the spin texture exhibits a periodic, nonlinear pattern, which deviates from the simple sinusoidal form typically observed in helical structures (Figure 1(c)). This anharmonic behavior of the spin configuration leads to the appearance of additional higher-order peaks.

**Figure 3.**
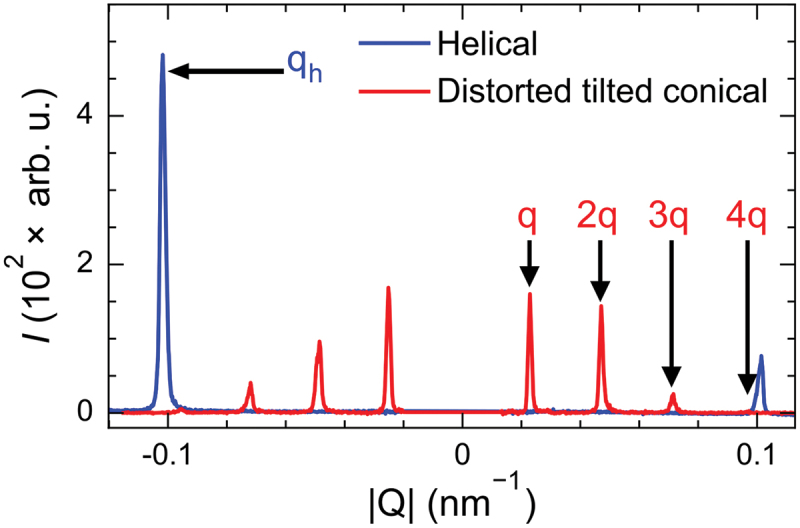
Comparison of the REXS intensity profile for helical (blue) and dTC spirals (red). The figure highlights a single peak ($q_{h}$) from the Friedel pair of the helical spirals at 0 T, and four peaks (q, 2q, 3q, and 4q), each from an equally spaced Friedel pair of the dTC at 45 mT, applied at an angle of $\phi =6^{\circ }$ to the [001] direction. The intensity peak corresponding to the structural Bragg peak is manually removed.

Figure 3 shows an exemplary intensity line profile of a CCD image containing the magnetic satellites from REXS in the *hk1* plane of the reciprocal space. The intensity profile shows a single Friedel pair ($\pm q_{h}$) for the helical spiral at zero applied magnetic fields that are governed by a weak cubic anisotropy [32]. For the dTC phase having a spiral modulation with non-linear spin rotation angles, four Friedel pairs (±q, ±2q, ±3q, ±4q) are identified at 8 K with $\mu_{0}H$ = 45 mT (see Figure 3). The propagation vector of the helical spiral, defined as $q_{h}=2\pi /\lambda_{h}$, is estimated to be around 0.1 nm${\;}^{-1}$ [5,32], yielding a helical spiral wavelength $\lambda_{h}\approx$ 60 nm. However, $q_{h}$ ≈4×q suggests that the dTC spiral phase has a significantly longer wavelength in real space, estimated to be around 240 nm at $\phi \;\;=6^{\circ }$ (see Appendix B for the rocking results).

**Figure 4.**
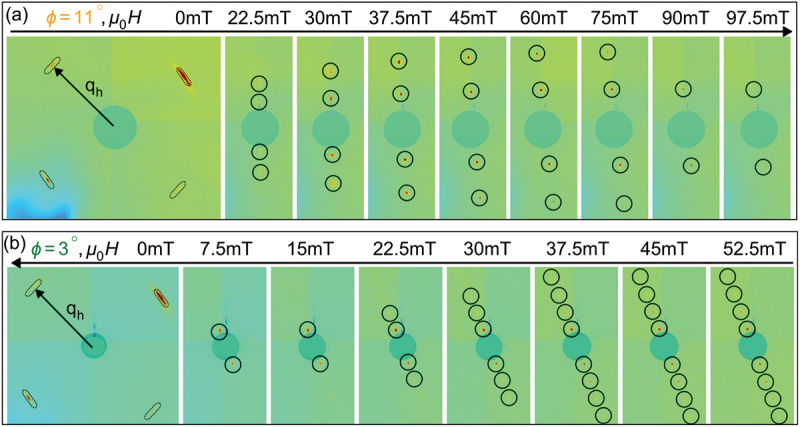
(a) and (b) Evolution of the dTC spiral phase under different applied magnetic fields with REXS at 8 K. (a) and (b) shows the REXS intensity patterns obtained for two different magnetic field sweep directions. $q_{h}$ indicates the helical propagation vector defined at 0 T. The circles highlight the positions of higher-order peaks of the dTC spiral phase. Note that the measurements shown in (a) and (b) have been carried out for two different magnetic field orientations with respect to the [001] axis represented as ϕ.

**Figure 5.**
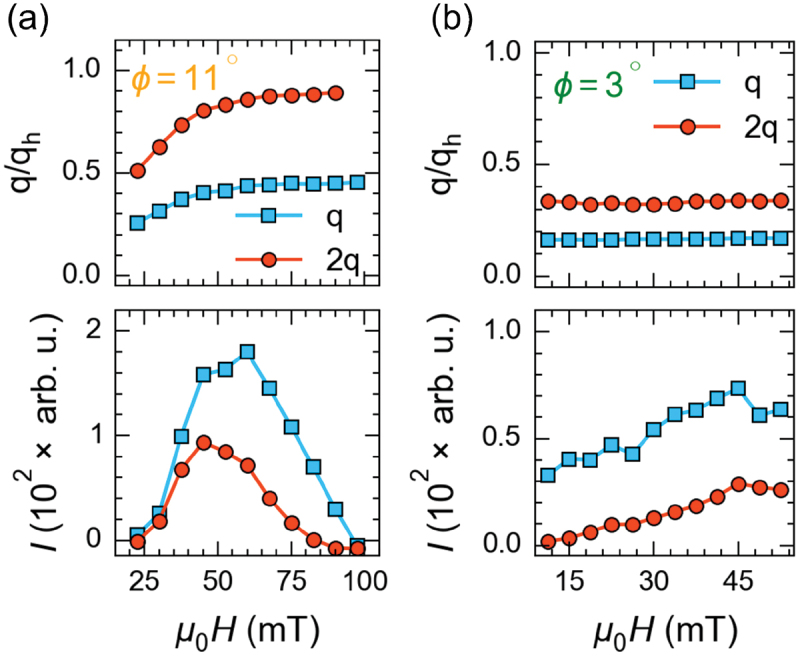
(a) and (b) show the change of both propagation vector and intensity of the first and second-order magnetic peaks (q and 2q) as a function of the applied magnetic field for ramping the magnetic field from zero to the field polarized state (a) and ramping the magnetic field to zero (b).

**Figure 6.**
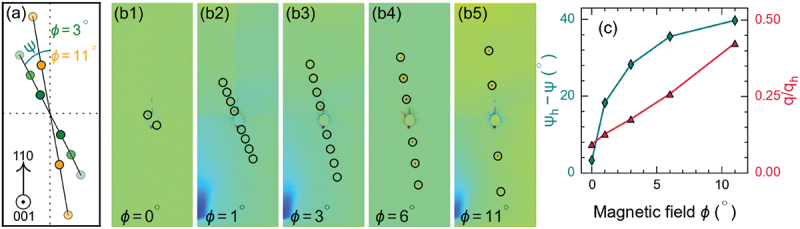
(a) Schematics depicting the reorientation of the dTC spiral when tilting the applied magnetic field at an angle ϕ. The reorientation angle ψ is defined as the in-plane rotation angle relative to the [110] crystallographic direction. (b) REXS intensity profile recorded at T≈ 8 K and $\mu_{0}H$ =45 mT applied at angle ϕ. (c) Change of reorientation angle ($\psi_{h}-\psi$) and normalized wave vector (q/q${\;}_{h}$) of the dTC spiral as tilt angle ϕ of the applied magnetic field. q${\;}_{h}$ and $\psi_{h}$ represent the wave vector and reorientation angle of the helical spiral measured at zero magnetic fields, respectively.

Figure 4(a,b) show the color maps of the REXS intensity in reciprocal space, captured in the hk1 plane under various applied magnetic fields using a CCD detector for different magnetic field sweep directions. At zero applied magnetic field, we observe four peaks corresponding to two helical domains, as illustrated in Figure 4(a). With an increase of the magnetic field, the intensity of these helical peaks diminishes, while the dTC peaks start to appear at a relatively low magnetic field strength ($\mu_{0}H\approx 22$) (see Appendix A). As the magnetic field is increased further, the wave vector and peak intensity of the dTC phase progressively increase until 60 mT, after which a steady decline in peak intensity and saturation of the relative change in wave vector leads to their complete disappearance at about 100 mT, indicating the transition to a field-polarized state, as depicted in Figure 5(a,b). Note that at smaller magnetic fields close to applied at an angle $11^{\circ }$ , the dTC peaks are fully aligned along the [110] crystallographic direction, as depicted in Figure 4(a). When increasing the applied magnetic field, the dTC phase tilts away from the [110] axis with an angle . A tilt angle $\psi =4.5^{\circ }$ is observed at 60 mT, as shown in Figure 6, indicating the deviation of the spiral propagation vector towards the easy axis due to the prevailing cubic anisotropy. A similar trend is observed for an opposite-field sweep direction at , with no change in the spiral wave vector, as the magnetic field is decreased from the field-polarized state to 0 T (cf. Figure 5(a,b)). Importantly, both helical and dTC phases were simultaneously observed from 15 mT to 7.5 mT while sweeping the magnetic field to zero (see Appendix A). At zero magnetic field, only four helical peaks are observed.

Recently, higher-order peaks have been observed in a strained Cu${\;}_{2}$OSeO${\;}_{3}$ characterized by a chiral soliton lattice with the modulation vector along the strain direction [1]. The chiral soliton lattice (CSL) typically forms in uniaxial (strained) chiral magnets, with a significant reduction in the q vector [1,40] when a magnetic field is applied perpendicular to the propagation vector. In contrast, our study reveals distinct behavior: no significant change in the q vector was observed when the field decreased from 50 mT to the helical phase, while an increase in the q vector was noted as the field increased from 0 T to 60 mT. This was accompanied by a consistent rotation in q space, suggesting the stabilization of a dTC state. These findings indicate that the observed phase does not correspond to a CSL but rather defines a unique distorted spiral state. Recently, a distinct surface state resembling the dTC phase was reported using SQUID-on-tip microscopy on the surface of a bulk Cu${\;}_{2}$OSeO${\;}_{3}$ crystal. This study identified a stripe surface state and a tilted spiral state in real space, with the stripe state exhibiting higher-order magnetic satellites through FFT analysis of the real-space images [41].

By tilting the magnetic field, the dTC phase reorients, and the number of observed higher-order peaks decreases in the reciprocal map space, as illustrated schematically in Figure 6(a). At $\phi \;=0^{\circ }$, the dTC phase is oriented along the [010] crystallographic direction, parallel to one of the observed helical domains at 0 T, resulting in $\psi_{\text{h}}-\psi \approx 0$ as shown in Figure 6(b1). As the tilt angle of the magnetic field, ϕ, increases the dTC phase starts to reorient towards the [110] axis (along the vertical line drawn in the schematics of the CCD image in Figure 6(a)), resulting in an increase of the normalized pitch wave vector (q/q${\;}_{h}$) and spiral reorientation angle $\psi_{\text{h}}-\psi$ (see Figure 6(c)). Importantly, a substantial increase in the wave vector of the dTC phase is observed as a function of applied magnetic field tilt angle ϕ, which shifts the higher-order peaks outwards compared to the center of the CCD image and reduces the number of observable higher harmonic peaks in the reciprocal space image (cf. Figure 6(b1-b5)). The linear increase in the wave vector (q/q${\;}_{h}$) with increasing tilt angle ϕ is due to the projection of the dTC phase onto the (110) plane. As the spiral tilts away from the magnetic field, it projects more clearly onto this plane at an angle less than 90 degrees.

A hexagonal single-domain skyrmion lattice is formed by three coplanar propagation vectors aligned 60${\;}^{\circ }$ with respect to each other [33]. Whereas, within a multi-domain skyrmion lattice, the six-fold symmetric peaks divide into multiple six-fold subsets that are simultaneously sampled by the wide X-ray beam [38,42].

**Figure 7.**
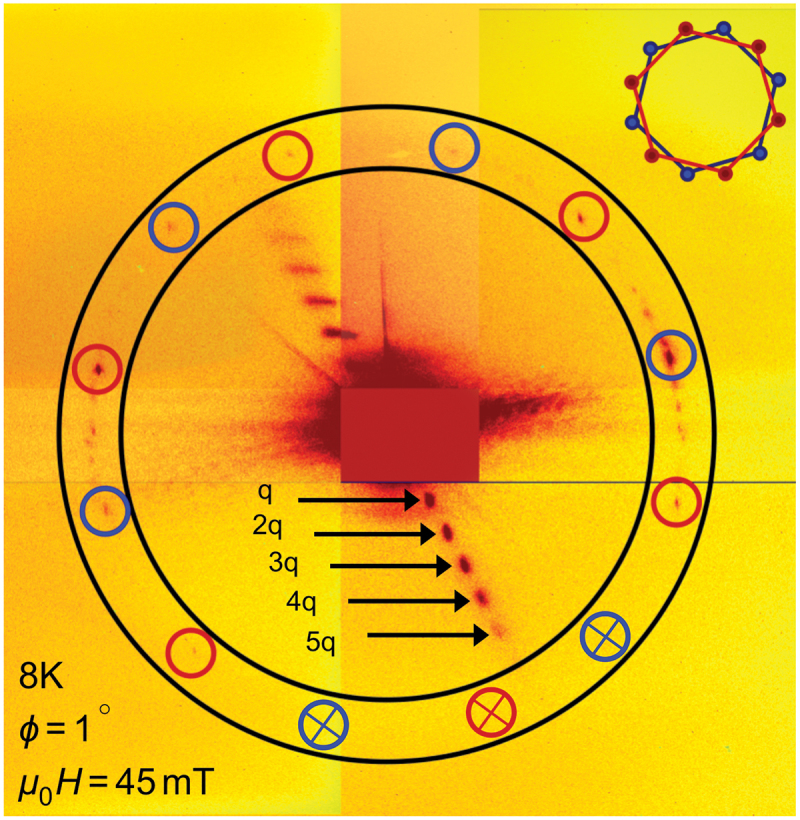
Reciprocal space map elaborating the coexistent dTC spiral and skyrmion lattice phases captured through CCD imaging at $\mu_{0}H$ = 45 mT and field angle ϕ = 1${\;}^{\circ }$ at 8 K. The red and blue circles highlight the peaks associated with two hexagonal skyrmion lattice domains. The inset shows the schematic diagram of two hexagons (red and blue) that are differently oriented w.r.t each other. Note that due to technical constraints, we did not observe magnetic satellites within the crossed circles.

Figure 7 reveals the reciprocal map of the skyrmion lattice state with six magnetic satellites linked to the structural Bragg peak. It demonstrates the coexistence of both dTC spiral and multidomain skyrmion lattice phases at $\mu_{0}H$ = 45 mT. The intensity pattern shown in Figure 7 is recorded in four instances with an exposure time of 1200 seconds for each around the Bragg peak to avoid intensity saturation within the CCD and later stitched together to show a full pattern for the skyrmion lattice. The area bounded by two concentric circles in Figure 7 marks the region where we anticipate observing a hexagonal scattering pattern of a skyrmion lattice [17]. The blue and red circles highlight the peaks with higher intensity and are considered as two hexagonal skyrmion lattices which are oriented differently (depicted in the inset) and appear as a multi-domain skyrmion phase. Moreover, in contrast to the representation in Figure 6(b) with $\phi \;=1^{\circ }$, prolonging the exposure time on the CCD leads to improved visibility of higher harmonic TC phase peaks up to the fifth order, as demonstrated in Figure 7 with arrows. When utilizing the REXS technique, its high sensitivity to the sample’s surface allows us to precisely identify the magnetic textures present near the surface. This capability highlights unique surface magnetic textures, which may differ from the bulk configuration due to surface anisotropy and surface Dzyaloshinskii–Moriya interaction [23], leading to the observation of distinctive distorted spin textures that are not present in bulk chiral systems [6,28].

## 4. Summary

We experimentally identified an ordered surface texture referred to here as distorted titled conical spiral (dTC) phase in an extended field region of the magnetic-phase diagram at low temperatures. The dTC phase points towards the presence of additional magnetic interactions playing an important role at the surface.

The dTC phase does not disappear with field cycling; instead, it coexists with the multidomain skyrmion phase populated by the field cycling protocol. The dTC and skyrmion phases show a strongly hysteretic behavior depending on the direction of the applied magnetic field sweep. Additionally, we examined the reorientation of the dTC phase by tilting the static magnetic field relative to the crystallographic direction of Cu${\;}_{2}$OSeO${\;}_{3}$. Notably, the dTC phase propagation vector follows the magnetic field in the ([001]–[110]) plane with a deviation angle ψ. We observed a nonlinear change in angle $\psi_{\text{h}}-\psi$ for magnetic field angle (ϕ) from almost zero degrees (at $\phi \;=0^{\circ }$) to about 40 degrees (at $\phi \;=11^{\circ }$) in the *hk1* plane. Furthermore, we recognized a linear increase of the normalized modulation vector $|\text{q}|/|{\text{q}}_{\text{h}}|$ by tilting the magnetic field with angle ϕ.

Our findings provide valuable insights into the periodicity and orientation of the rarely studied dTC phase at the surface of Cu${\;}_{2}$OSeO${\;}_{3}$ crystals with a remarkably long period of approximately 240 nm. Resolving the dTC phase and other magnetic textures at the surface highlights the importance of REXS for identifying and engineering chiral surface twists. This research opens new avenues for investigating the behavior of these intricate spin textures under various conditions, contributing to the broader knowledge of chiral magnetic materials.

## Figures and Tables

**Figure 8.**
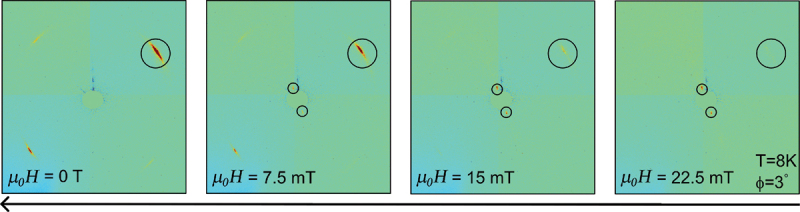
Color maps of REXS intensity in reciprocal space for four different magnetic fields while reducing the field to zero at 8K with $\phi \;=3^{\circ }$. The black arrow shows the magnetic field sweeping direction. At 22.5 mT, two peaks associated with the dTC are observed. Between 15 mT and 7.5 mT, both dTC (enclosed by small circles) and helical (enclosed by a large circle) magnetic satellites are visible. Only the four magnetic satellites corresponding to the helical spiral state are observed at zero magnetic fields.

**Figure 9.**
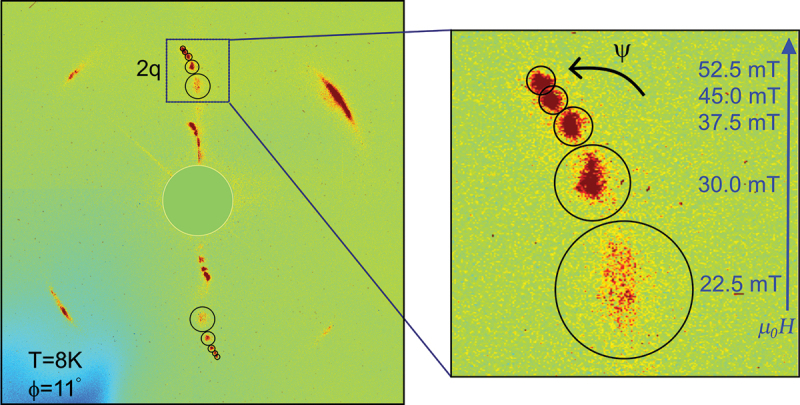
Stacked image of the REXS measurements for the magnetic field sweeping from 0 to 52.5 mT in 7.5 mT increments at $\phi \;=11^{\circ }$. The four magnetic satellites correspond to the helical state, and when the magnetic field reaches 22.5 mT, the first and second harmonic peaks (q and 2q) of the dTC phase emerge with a spread intensity pattern (surrounded by a larger circle). As the magnetic field increases, these peaks move outward with respect to the Bragg peak, accompanied by a tilt. The evolution of the second harmonic peaks (2q) indicates the tilting of the spiral propagation vector with increasing magnetic field strength.

**Figure 10.**
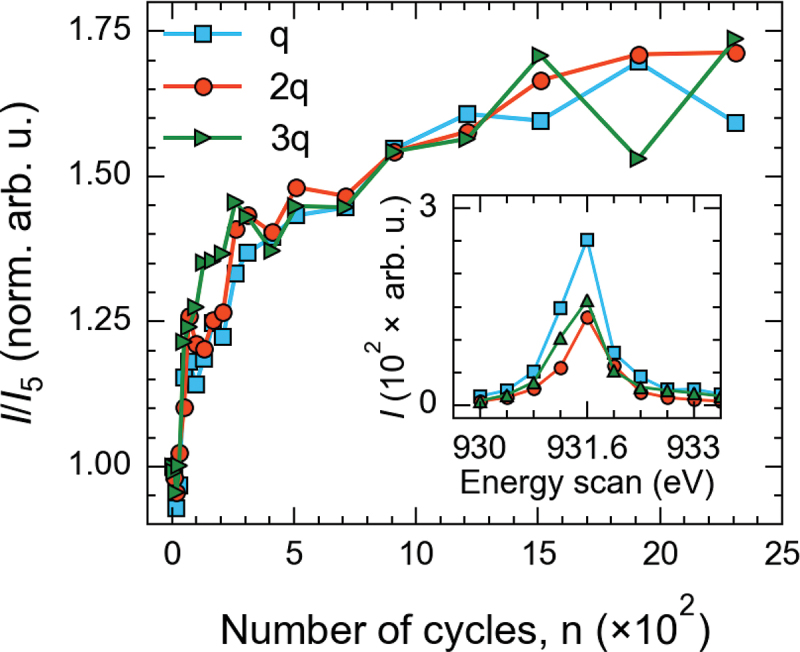
Normalized peak intensity ($I/I_{5}$) of the peaks in the dTC phase as a function of number of cycles n of the static applied magnetic field in the range of 52 mT to 45 mT at T≈ 8 K. Where $I_{5}$ represents the peak intensity measured after five field cycles. After field cycles n, each data point is recorded at $\mu_{0}H$ = 45 mT. The inset shows the intensity amplitude for different energies in the vicinity of L${\;}_{3}$ edge (931.8 eV) for the first, second, and third higher-order peaks of the dTC.

**Figure 11.**
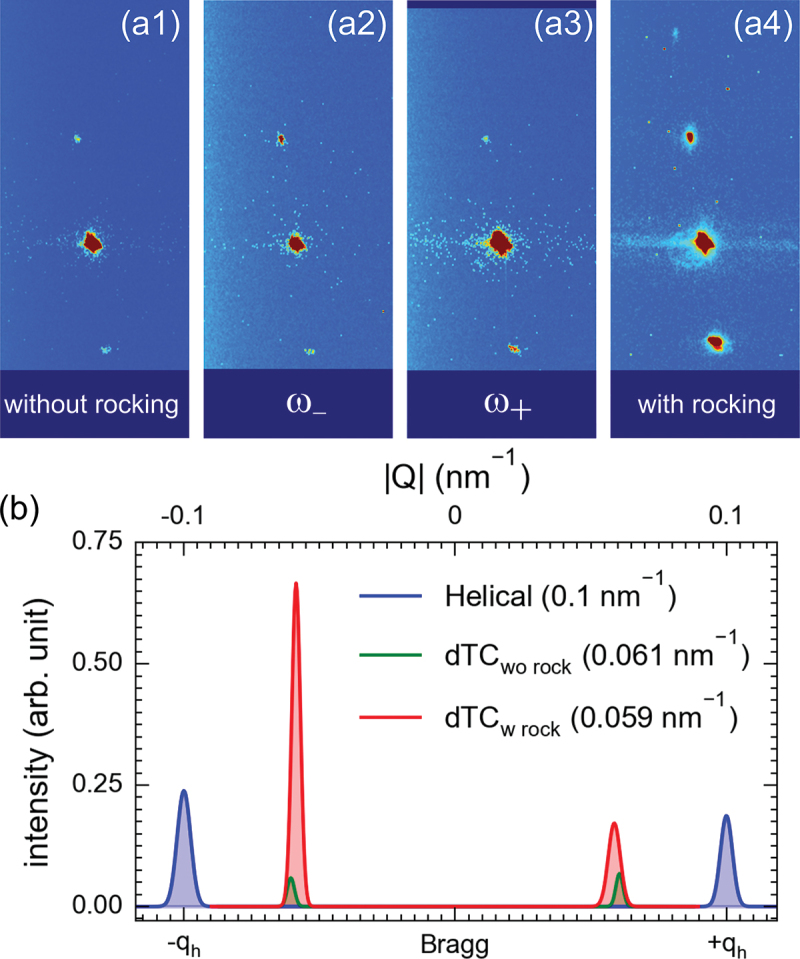
(a1–a4) present typical REXS images of the distorted tilted conical (dTC) phase for the non-rocked sample, left and right sides of rocking ($\omega_{-}$ and $\omega_{+}$), and the integrated rocking image respectively. (b) Highlights the impact of rocking on the dTC magnetic satellites, providing a comparison with the helical vector $q_{h}$. Note that the plot represents the data fitted with a Gaussian function, and the error bars are negligible.

**Figure 12.**
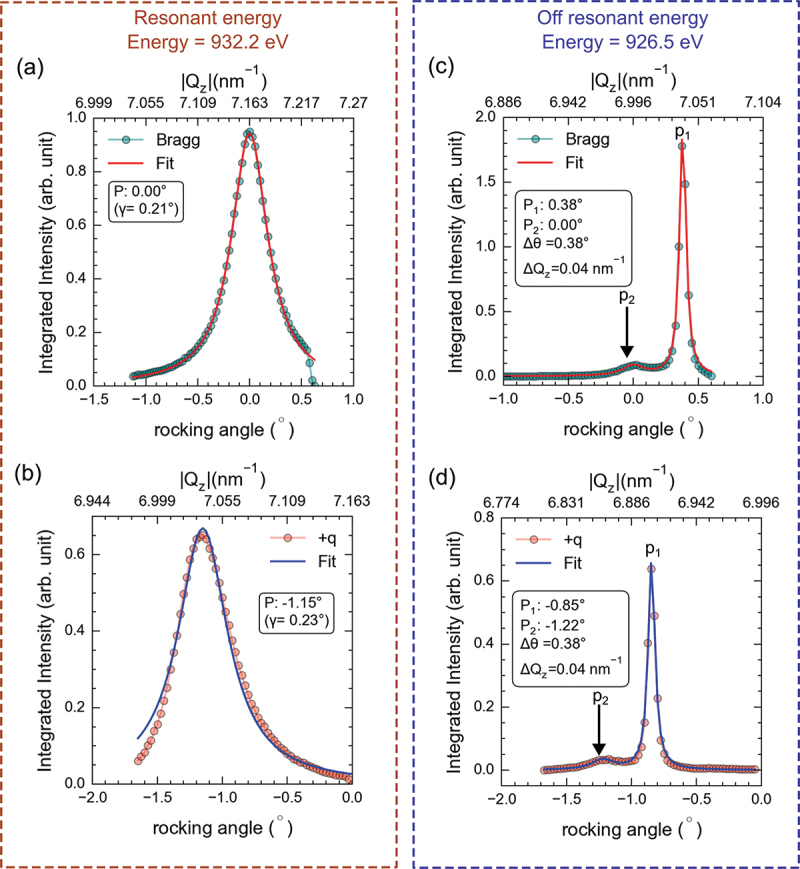
(a) and (b) display the integrated intensity of the Bragg peak and the first harmonic magnetic satellite of dTC as a function of the rocking angle at the resonant energy L${\;}_{3}$ edge (932.2 eV) fitted by a Lorentzian function (FWHM = 2γ). Similarly, (c) and (d) show the corresponding data at the off-resonant energy (926.5 eV) where P${\;}_{1}$ and P${\;}_{2}$ show the 001 peak and modulation along Qz component, respectively.

## Appendix A

Figure 8 shows the REXS intensity pattern while the magnetic field is swept to zero at an angle of $\phi \;=3^{\circ }$ at 8 K. We expect four magnetic satellites in the *hk1* plane corresponding to the helical phase at low magnetic fields. When the magnetic field reaches 22.5 mT, these four peaks start to appear with low intensities, becoming more prominent as the magnetic field decreases to zero (see the large circle in Figure 8). In our study, we observed these helical satellites along with the coexistence of dTC (highlighted by small circles) within a narrow magnetic field range from 22.5 mT to 7.5 mT, but only when the magnetic field is swept from field-polarized to zero.

To study the evolution of the dTC phase under varying magnetic field strengths, we compiled REXS intensity patterns from 0 T to 52.5 mT (9 instances), as shown in Figure 4 of the main manuscript. These patterns allow us to observe how the spiral structures respond to different magnetic field strengths. Initially, from zero magnetic field up to 15 mT, we observed only the four magnetic satellites associated with the helical state. This indicates that at lower magnetic fields, the system maintains a helical configuration without any noticeable transition to other states. As the magnetic field increases beyond 22.5 mT, the peak intensities of the dTC phase become apparent. This marks a significant transition point where the magnetic field strength is sufficient to change the configuration of the spiral structures. At 22.5 mT, the q and 2q peaks align with the $\left\langle 011\right\rangle$ direction, forming a broad intensity spot highlighted by a large circle in Figure 9. With further increases in the magnetic field, the propagation vector of the dTC continues to gradually tilt away from the $\left\langle 011\right\rangle$ direction ($\psi \approx 5^{\circ }$). This tilting is accompanied by changes in the modulation q vector, as illustrated in Figure 5 of the main manuscript. For magnetic fields above 22.5 mT, the peak intensities become narrower, as indicated by the smaller circle in Figure 9. This narrowing suggests that the spiral structures are becoming more defined and possibly more stable under higher magnetic field strengths. This specific behavior has been observed only when sweeping the magnetic field from zero to a field-polarized state. The process of gradually increasing the magnetic field allows us to capture the transitions and reorientations of the spiral structures in detail. Moreover, from 52.5 mT to 100 mT, no changes were observed in the modulation vector or the propagation vector of the dTC. This plateau indicates that the spiral structures have reached a stable configuration that does not further evolve with increasing magnetic field strength within this range.

Figure 10 indicates an increase in the intensity of the peaks observed in the dTC phase with magnetic field cycling in the range of $\mu_{0}H_{\mathrm{low}}$ 45 mT to $\mu_{0}H_{\mathrm{high}}$ = 52 mT within the spiral magnetic phase. The field cycling impact on peak intensities of |q|, |2q|, and |3q| (first, second, and third harmonic, respectively) are shown after normalizing the corresponding intensities with the intensity observed after five cycles. The inset in Figure 10 shows the intensity amplitude of the peaks as a function of X-ray beam energy that confirms the highest intensity occurs for the energy around L${\;}_{3}$ edge. Notably, the intensity starts at a baseline normalized value of 1 and demonstrates an initial rapid increase, reaching almost 1.5 after 500 cycles. Thereafter, the intensity exhibits a gradual rise, ultimately approaching a saturation point after 1500 cycles. Note that the intensities shown in Figure 10 do not represent integrated intensity. Therefore, the observed increase in the peak intensity with field cycling can be just a relative increase at the measured spot.

## Appendix B: Rocking sample

In resonant elastic X-ray scattering (REXS) experiments, rocking scans are commonly used to enhance the resolution of the scattering intensity and improve the accuracy of determining the propagation vector q in chiral magnets such as Cu${\;}_{2}$OSeO${\;}_{3}$. By scanning across a range of angles, rocking allows for a more comprehensive measurement of the intensity distribution of both Bragg and magnetic satellite peaks, reducing uncertainties related to peak positions and shapes. Without rocking, the CCD captures only a single projection of the scattered intensity, which may lead to a slight underestimation of peak intensities and a less complete mapping of reciprocal space, particularly affecting weaker magnetic satellite peaks. Additionally, the absence of rocking can result in broader peaks due to projection effects, making the precise determination of their positions and intensities more challenging. These factors can introduce small systematic errors in the extracted wave vector q, potentially leading to slight deviations from the expected value. Moreover, since REXS is sensitive to both surface and bulk effects, non-rocking measurements might be more influenced by surface states rather than the bulk magnetic structure at resonant energy. Nevertheless, despite these potential drawbacks, measurements performed without rocking still retain significant value. The key features of the magnetic scattering, such as the presence and approximate positions of the magnetic satellite peaks, remain observable and analyzable. While the precision of peak intensity and width measurements may be slightly reduced, the overall conclusions regarding the magnetic structure and helical modulation are not fundamentally compromised. Therefore, although rocking scans improve accuracy, the non-rocked measurements in this study remain valid and sufficient for extracting meaningful information about the system’s magnetic texture and propagation vector evolution by canting magnetic field direction away from the [001] direction.

We conducted two measurements – one with rocking and one without – at a fixed magnetic field and a temperature of approximately 8 K to compare the impact of rocking on dTC peaks. As shown in Figure 11(a4), the peak intensities are more pronounced when using the rocking technique (cf. Figure 11(a1,a4)). Figure 11(a2,a3) corresponds to the single-shot CCD image at the left ($\omega_{-}$) and right side ($\omega_{+}$) of the rocking curve, respectively, and show the unbalanced magnetic satellite intensities around the Bragg peak. Figure 11(b) compares the dTC peak satellites, revealing a change in the magnitude of the q-vector by approximately 0.002 nm${\;}^{-1}$ indicating an error of about 3.28% for the cases without rocking in this study. This highlights the necessity of rocking scans for quantitative analysis of dTC spin textures in reciprocal space. However, the primary focus of this study is on the presence of the dTC phase and its evolution with the canting of the magnetic field direction from [001] to [111].

The rocking scan experiments were performed with the 2θ angle fixed at 96.5${\;}^{\circ }$, and the rocking ω angle is varied within ±1.5${\;}^{\circ }$ in 0.025${\;}^{\circ }$ steps while the sample and magnet are coupled. Due to the high-resolution geometry (the CCD-sample distance), the rocking range is limited to ±1.5${\;}^{\circ }$, as larger angles would cause all satellite peaks to move out of the CCD’s field of view which is defined by the instrument and sample geometries.

The rocking curve was measured for an arbitrary magnetic field direction relative to the [001] direction at both the resonant energy (Figure 12(a,b)) and below the resonant energy (Figure 12(c,d)). For each of these energies, the rocking scan was conducted for both the structural Bragg peak and the dTC peak. In the case of the Bragg peak rocking, the measurement was performed using a 0.1 s exposure time without the beam stopper. Conversely, for the dTC rocking curve, the beam stopper covered the Bragg peak to prevent overexposure of the CCD, with an exposure time of 10 s and the motorized beam stopper position was adjusted for each scan point. Notably, since the penetration depth at the resonant energy peak is lower than at off-resonant energy, the measurement is sensitive to the surface, approximately within tens of nanometers. In contrast, the penetration depth is significantly higher at an energy of 926.5 eV, referred to as the pre-edge (see Ref [25] for detailed sampling depth in the main manuscript).

With the rocking curve at the resonant energy (Figure 12(a,b)), one can immediately observe from the Gaussian profile that there is no modulation along the [001] crystal axis. This indicates that the REXS measurement is mostly surface-sensitive, as also discussed in the main manuscript. However, at the pre-edge energy, the rocking curve reveals a sharp 001 peak accompanied by a shoulder feature on its left side (Figure 12(c,d)). This suggests a modulation in the out-of-plane (z) direction with the magnitude Q${\;}_{z}$ of ≈ 0.041 nm${\;}^{-1}$, which is not seen at the resonant condition due to the limited penetration depth. Notably, the observed shift in the 001 peak P${\;}_{1}$ aligns well with the change in X-ray energy in the Bragg condition, calculated as $\Delta \theta =|\theta_{\mathrm{res}}-\theta_{\mathrm{off}-\mathrm{res}}|=0.39^{\circ }$. The total scattering vector Q${\;}_{\mathrm{tot}}$ can be derived from the geometrical sum of Q${\;}_{\mathrm{xy}}$ and Q${\;}_{z}$ components as Q${\;}_{\mathrm{tot}}=[(Q_{xy}^{2}+Q_{z}^{2}){]}^{1/2}=0.0718\;\;nm^{-1}$. This is unexpectedly small compared to a bulk TC (0.095 nm${\;}^{-1}$, see the main manuscript Ref [7]), indicating a distortion that influences the magnetic texture. This also adds a value compared to the recent imaging work in Ref [41], because the authors assumed the fixed $Q_{\mathrm{tot}}$ value taken from the bulk SANS while imaging only the in-plane component. Such distortion likely contributes to the emergence of higher-order harmonics, further modifying the scattering profile as discussed in the main manuscript.
